# Contracting the gap: The effects of identity gaps on the psychological adaptation of Taiwanese university students

**DOI:** 10.1371/journal.pone.0349289

**Published:** 2026-05-19

**Authors:** Jiaying Lan, Kartini Aboo Talib @ Khalid, Shazlin Amir Hamzah, Peng Kee Chang

**Affiliations:** 1 Institute of Ethnic Studies, Universiti Kebangsaan Malaysia, Selangor, Malaysia; 2 College of Humanities, Xiamen Huaxia University, Xiamen, China‌‌; Tianjin University, CHINA

## Abstract

With the rapid internationalization of higher education, patterns of student mobility have moved beyond the traditional intercultural model of East-to-West migration. Mainland China has become a major destination for international students, and the number of Taiwanese university students studying there has steadily increased recently. This trend underscores a significant research gap regarding the psychological adaptation of students engaged in intra-cultural mobility within Eastern cultural contexts. This study extends identity gap research, a core concept of the Communication Theory of Identity (CTI), to a less explored intra-cultural educational mobility framework. It empirically examines the associations among Personal Enacted Identity Gap (PEIG), Personal Relational Identity Gap (PRIG), and Personal Communal Identity Gap (PCIG), as well as their associations with the psychological adaptation of Taiwanese university students in mainland China, with the aim of identifying the identity construct most consistently associated with psychological adaptation. A cross-sectional survey was conducted with 425 Taiwanese university students in Fujian Province. Data were analyzed using partial least squares structural equation modeling (PLS-SEM), two alternative models, and PLS-IPMA. The findings show that identity gaps are negatively associated with psychological outcomes in an Eastern cultural context, and that the PCIG is the construct most consistently negatively associated with psychological adaptation. These findings extend the understanding and applicability of the CTI and highlight the cultural sensitivity of identity gaps across different contexts. This study also provides important implications for policymakers and educational administrators seeking to design and implement strategies to promote the psychological adaptation of Taiwanese university students in mainland China.

## 1. Introduction

Mainstream research on international students’ cross-cultural adaptation has predominantly focused on migration from developing to developed countries, usually within Western contexts [1,2]. Limited attention has been given to the mobility of international students toward developing countries, particularly those situated within Eastern cultural contexts. More notably, research examining migration and adaptation within the same cultural sphere, such as regions shaped by Confucian traditions, is especially scarce. Recently, mainland China has emerged as a dynamic and increasingly influential destination for international student mobility, continuously attracting a substantial number of students from Korea, Japan, Hong Kong, Macau, and Taiwan [3,4]. Unlike cross-cultural transitions between culturally distant East and West contexts, educational migration within the Confucian cultural sphere may involve more nuanced yet distinct identity challenges and adaptation difficulties [5]. However, these intra-cultural adaptation experiences have so far received limited scholarly attention. Among these groups, Taiwanese university students represent a particularly underexamined population. Recent statistics indicate a steady increase in the number of Taiwanese students pursuing higher education in mainland China [6,7]. This trend makes the adaptation of Taiwanese university students in mainland China an issue that warrants urgent scholarly attention.

Despite shared cultural roots, Taiwanese students in mainland China encounter significant adaptation challenges. These difficulties stem from a confluence of factors, including distinct historical trajectories, divergent sociopolitical systems, and different socialization processes. Collectively, these influences have contributed to variations in social values and communicative norms [8,9]. Such discrepancies often manifest as core sources of identity tension during their transition.

The important role of identity in the adaptation process has been well established in the literature [10,11]. At the same time, prior research has shown that the processes of identity adjustment and transformation are often complex. Owing to differences in language, culture, customs, and social norms, migrants frequently encounter identity conflicts. Within the framework of the communication theory of identity (CTI), such conflicts are conceptualized as identity gaps [12,13]. Existing research on identity gaps among migrant and sojourner populations has predominantly focused on psychological outcomes, such as distress and life satisfaction [11,14–16]. However, no study has directly linked identity gaps to psychological adaptation. In addition, prior research on identity gaps in mobile populations has largely been conducted within Western cultural contexts. Given the close relationship between identity and cultural environments, as well as the substantial differences between Eastern and Western cultures, examining the cultural sensitivity of identity gaps in Eastern contexts represents a significant research gap. Furthermore, previous studies have mainly focused on the effects of personal, relational, and enacted identity gaps [15,17,18], whereas the present study places particular emphasis on the personal communal identity gap (PCIG). By examining the interrelationships among different types of identity gaps, this study also addresses the limitation of prior research that has largely focused on the effects of identity gaps on outcome variables while overlooking the relationships among identity gaps themselves.

By introducing the CTI into the context of intra-cultural adaptation in Eastern societies, this study not only validates the theory’s cross-cultural applicability and cultural sensitivity but also extends its scope within the field of international student research. Using Taiwanese university students in mainland China as a unique case, this study provides an in-depth analysis of the dynamic evolution of their identity negotiation and the adaptation challenges they encounter. By establishing a logical intervention pathway in which reducing identity gaps contributes to improved psychological adaptation, the study not only offers practical recommendations but also provides empirical support for policymaking and educational governance in China and other Eastern cultural contexts.

## 2. Literature review and hypotheses development

### 2.1. Definition and development of the communication theory of identity

The CTI was first proposed by Michael L. Hecht and colleagues [12]. In essence, CTI posits that identity is fundamentally a communication process. Identity is not only expressed through communication but is also formed and continuously reshaped by communication itself. Within CTI, this interactional identity consists of four layers.

The personal frame refers to the individual’s self-concept, self-image, or felt sense of self, namely one’s internal and external self-evaluations. It serves as a source of personal expectations and motivations and reflects characteristics, beliefs, and feelings about oneself [13]. The enactment frame emphasizes the communicative expression of identity. It concerns how individuals perform or manifest identity in interaction through messages, behaviors, symbols, and roles enacted in communication, all within given social, cultural, physical, and environmental constraints [19]. The relational frame arises from relationships with others and has multiple dimensions. It includes identity in relation to others; identity ascribed by others; and identity formed through relationships. The communal frame represents the collective layer of identity tied to group membership and social categories. This refers to identities ascribed by the larger community or culture to the individual and shared with a group, such as national, ethnic, or religious identities, or membership in a profession or demographic category [12]. Communal identity reflects the traits, values, and identifications derived from being part of communities and from broader cultural narratives about those communities.

According to the CTI, identity layers are interpenetrated and evolve through ongoing social interactions [12]. The theory posits that dialectical tensions may exist among these layers, which can sometimes be contradictory while at other times mutually reinforcing. When the layers are consistent and coherent with one another, identity remains in a state of harmony. However, when discrepancies or conflicts occur between layers, tension arises [13], defining such tensions as identity gaps. Their research initiated a new direction of scholarship, focusing on interpenetration and dynamic interplay among identity layers. The present study specifically focuses on three types of identity gaps: the PEIG, the PRIG, and the PCIG.

### 2.2. Identity gaps among Taiwanese university students

The PEIG refers to the difference between how individuals express themselves and how they perceive themselves [20]. For Taiwanese students in mainland China, this gap can arise when they suppress their opinions in reserved classroom settings or practice self-censorship due to perceived political constraints, thus creating a divergence from their authentic selves [21,22]. The PRIG is the inconsistency between one’s self-concept and the identities ascribed by others [20]. This often stems from cultural misunderstandings; for instance, informal classroom behaviors accepted in Taiwan, such as casual attire, might lead mainland peers to form negative stereotypes (e.g., viewing them as undisciplined) that conflict with the students’ own self-perceptions. The PCIG refers to the disconnect between how individuals define themselves and how the social groups to which they belong are defined [14,20]. For example, this gap can emerge from differing cultural definitions of a “successful” student. For many mainland students, success is tied to collectivist values such as family honor and quantifiable academic achievements. In contrast, Taiwanese students may prioritize more individualistic goals like inner satisfaction and diverse experiences. Consequently, when a Taiwanese student’s focus on extracurriculars results in a lower class ranking, mainland peers may perceive them as “unsuccessful,” creating a direct conflict with the student’s own belief that their varied experiences constitute a successful university life.

### 2.3. Identity gaps and psychological adaptation

It is widely established that cross-cultural adaptation comprises two dynamic dimensions, psychological and sociocultural [23–25]. Given that existing research on identity gaps has primarily focused on psychological outcomes, the present study follows this line of work and adopts the model proposed by Searle and Ward [23] to focus specifically on psychological adaptation. Psychological adaptation reflects the psychological changes that individuals undergo during the cross-cultural process, indicating whether they feel comfortable and satisfied or anxious and maladjusted in a new cultural environment [26,27]. In the literature on immigrants adapting to Western cultural contexts, extensive research has demonstrated that persistence in maintaining one’s original identity or strategically modifying it can exert varying degrees of influence on integration into a new cultural environment. For instance, researchers found that a strong ethnic identity promotes greater self-esteem and reduces depression among Chinese immigrant youth in Canada [28]. Flexible approaches, such as adopting hybrid or alternating identity styles, correlate with improved adaptation and decreased distress [29,30]. More recently, scholarly attention has gradually expanded to students engaged in intra-cultural mobility within Eastern cultural contexts. For instance, a stronger Chinese national identity predicted more positive psychological adaptation among Macao students in mainland China [10,31]. This evidence confirms that identity remains a critical factor influencing psychological adaptation, even within a shared cultural sphere.

Research on Taiwanese populations has demonstrated that their adaptation process in mainland China is also accompanied by unique identity-related challenges. Tang et al. [8] found that although Taiwanese residents in mainland cities expressed a certain level of identification with and willingness to participate in their host communities, their actual behaviors did not align with such attitudes. This inconsistency between identity and behavior resulted in an unstable state of identity, which constitutes a central difficulty in their deeper integration into mainland society. Similarly, Chen et al. [32] described Taiwanese individuals living in mainland China as “New Mobile Citizens.” These individuals frequently travel between cities across the Taiwan Strait and commonly experience a sense of rootlessness or displacement. The study further indicated that their psychological identification was primarily oriented toward practical benefits related to work and daily life, while at the cognitive and identity levels they lacked intrinsic motivation to enhance their sense of closeness to or identification with mainland China. As a result, they often demonstrated a “pragmatic sojourner” mindset, which poses enduring challenges for long-term adaptation. Overall, these studies highlight that the adaptation of Taiwanese populations in mainland China typically involves inconsistent and ambivalent identity issues. Given the increasing enrollment of Taiwanese students in mainland Chinese universities, addressing the research gap concerning their specific identity-related and adaptive challenges is of growing empirical importance.

The potential association between identity gaps and individual mental health outcomes has drawn increasing scholarly attention [33]. Empirical research has primarily concentrated on the personal enacted and personal relational identity gaps, which closely correlate with negative psychological outcomes. These two types of gaps directly reflect discrepancies in communicative behavior and in ascriptions by others, thereby illustrating how self, communication, and relationships constitute interrelated aspects of identity and provide a unique integrative approach to the study of identity and social relations [13]. Subsequent studies have extended the scope of inquiry to more complex and macro-level layers of identity, such as the personal–communal and communal–communal identity gaps [14,18,34]. In terms of research populations, early studies investigated the negative psychological outcomes of identity gaps among ethnic minority groups such as Korean Americans [20,35] and Latinos in the United States [15]. Later research expanded to populations undergoing significant identity transformations or those whose identities were challenged by dominant societal values, including military veterans [16], refugees [33], individuals with disabilities or chronic illnesses [36,37], and LGBTQ groups [38]. A substantial number of studies on adolescents, students, and international students have further confirmed the negative predictive role of identity gaps for outcomes such as well-being and satisfaction. For example, Shin and Stephenson [15] found that wider identity gaps intensified depressive symptoms and substance use among Mexican American adolescents. Ramsey et al. [17] reported that students’ self-sabotaging behaviors increased their identity gaps, which in turn reduced their willingness to seek help from teachers. Amado et al. [11] showed that international students in the United States experienced an expansion of the personal–enacted identity gap due to acculturative stress, which subsequently led to depressive symptoms. Daniels and Rittenour [39] found that among female international students studying, working, and socializing in the United States, the personal–relational identity gap negatively predicted communication satisfaction. Similarly, Kim et al. [40] focusing on international students undergoing cross-cultural adjustment in the United States, found that social networking site (SNS) affordances played a dual role in the management of identity gaps, yet wider identity gaps consistently predicted poorer cross-cultural adjustment. Overall, existing research has reached a clear consensus: the greater the identity gaps, the more pronounced the negative psychological outcomes for youth and student populations. However, this body of research has focused on general psychological outcomes without explicitly linking identity gaps to psychological adaptation. Moreover, these investigations have been predominantly conducted in non-Eastern and non-intracultural settings.

Therefore, the following hypotheses are proposed:

H1a: The PEIG is negatively associated with psychological adaptation.
H1b: The PRIG is negatively associated with psychological adaptation.
H1c: The PCIG is negatively associated with psychological adaptation.

Given that the CTI posits that identity is created and maintained primarily through communication and views communication as the enactment of identity, communication becomes involved in identity when individuals interact with others, detect how others view them in communication, and internalize those views. From this communication based perspective, the gap between personal identity and enacted identity, namely PEIG, can be understood as an intrapersonal tension because it originates at the starting point of interaction, that is, self-expression. By contrast, the gaps between personal identity and the relational and communal layers, namely PRIG and PCIG, are forms of interpersonal tension that arise through individuals’ interaction with the external world and are formed as individuals detect and internalize the views of others [13,41,42]. Accordingly, in terms of the formation mechanism of identity gaps within a single communicative cycle, PEIG represents a more proximal identity gap produced by communication, whereas PRIG and PCIG represent more distal identity gaps. Moreover, because CTI posits that the different layers of identity are interpenetrated, this interpenetration may imply that a gap arising between two layers can influence other layers and the discrepancies among them, thereby generating a dynamic process of identity misalignment. It can therefore be deduced that when inconsistencies occur between an individual’s personal identity and the ascriptions of others, that is, when a PRIG occurs, one possible explanation is that the individual fails to effectively communicate authentic thoughts or enact behaviors consistent with the self-concept. Such ineffective or distorted expressions may lead interaction partners to misinterpret the individual’s authentic self, thereby forming and intensifying the PRIG.

To date, a large proportion of empirical studies on identity gaps have concentrated on dyadic interpersonal contexts, such as student–teacher, romantic partners, or grandparent–grandchild relationships, with particular emphasis on the personal–enacted–relational core triad [38,43], and have found a significant positive correlation between PEIG and PRIG. [13,41–43]. In contrast, relatively little attention has been given to the communal layer gap. However, because communal identity is itself constructed from an interwoven set of social relationships, the relational identity formed through small-scale interpersonal communication is inherently embedded in, and impacted by, the broader interpersonal networks that define communal identity. Accordingly, it is reasonable to posit that both PEIG and PRIG may exert a positive influence on the PCIG.

Accordingly, the following hypotheses are formed:

H2a: PEIG is positively associated with PRIG.
  H2b: PEIG is positively associated with PCIG.
  H2c: PRIG is positively associated with PCIG.
  H3a: PRIG mediates the negative association between PEIG and psychological adaptation.
  H3b: PCIG mediates the negative association between PEIG and psychological adaptation.
  H3c: PCIG mediates the negative association between PRIG and psychological adaptation.

## 3. Methodology

This study was approved by the Research Ethics Committee of the Universiti Kebangsaan Malaysia and adhered to the Declaration of Helsinki. Written informed consent was obtained electronically from all participants. Before accessing the questionnaire, participants were informed of the study’s purpose, benefits, risks, and data usage and indicated their voluntary agreement by clicking a confirmation button. The survey was conducted anonymously, and no minors were included.

### 3.1. Measurement instrument

This study employed a structured questionnaire to measure the constructs relevant to the research questions. All scales were adapted from previously validated instruments, with modifications made to fit the specific context of the study. For detailed measurement items, refer to the S1 Appendix file. All items were measured using a seven-point Likert scale ranging from 1 (“Strongly disagree”) to 7 (“Strongly agree”). The questionnaire consisted of two sections. The first section collected respondents’ demographic information, and the second section included scale items corresponding to the study variables.

The Personal Enacted Identity Gap (PEIG) was measured using the scale developed by Jung [18]. In this study, the scale was used to assess the perceived difference among Taiwanese university students in mainland China between their self-view and the self expressed in communication with friends from mainland China. The scale consists of six items, for example, “I often hide some aspects of myself in communication with my friends from mainland China.” The Cronbach’s α coefficient for this scale was 0.890, indicating good reliability.

The Personal Relational Identity Gap (PRIG) was also adapted from the scale developed by Jung [18]. This scale was used to measure the perceived difference among Taiwanese university students in mainland China regarding their self-view compared to how they believe their friends from mainland China perceive them in communication. The scale consists of seven items, for example, “I feel my friends from mainland China stereotype me.” The Cronbach’s α coefficient for this scale was 0.921, indicating excellent reliability.

The scale developed by Murray and Kennedy-Lightsey [14] was modified to measure the Personal Communal Identity Gap (PCIG). In this study, the scale was used to assess the perceived difference among Taiwanese university students in mainland China between their self-view and their perception of how the groups to which their friends from mainland China belong see them in communication. The scale consists of seven items, for example, “I feel embarrassed that I am part of the community that my friends from mainland China belong to.” The Cronbach’s α coefficient for this scale was 0.847, indicating good reliability.

Finally, this study used the eight-item scale developed by Geeraert and Demes [44] to measure the psychological adaptation of Taiwanese university students in mainland China. An example item is, “I feel out of place when I do not fit into the culture of mainland China,” which is a reverse-coded item. The Cronbach’s α coefficient for this scale was 0.887, indicating good reliability. For detailed measurement items, refer to S1 Appendix file.

A pre-test was conducted prior to the main survey. Specifically, three experts in communication and anthropology, one each from Taiwan, mainland China, and Malaysia, were invited to evaluate the questionnaire in terms of face validity, content validity, and cultural appropriateness. Based on their feedback, minor revisions were made to the items. As the adapted measurement scales were originally in English, and given that the target population consisted of Taiwanese students whose mother tongue is Chinese, a bilingual Chinese–English instrument was developed. The principal investigator, who was proficient in both languages and had previously studied in Taiwan as an exchange student, translated the original English items into Chinese and localized the terminology into Taiwanese Mandarin. The fourth author then back-translated all scales into English to verify the accuracy of the translation. In addition, a bilingual teacher from a university in mainland China reviewed both the translated and back-translated versions of the scales to further ensure their semantic equivalence [45].

### 3.2. Study sites and data collection

This study employed a cross-sectional online survey design, and data were collected in Fujian Province. Fujian is the only provincial-level “demonstration area” designated by the Chinese government for cross-strait integration and development, with explicit policy attention devoted to promoting the integration of Taiwanese residents into local study, work, and daily life. These institutional arrangements make Fujian an empirically meaningful site for examining adaptation processes under intensified cross-strait mobility. In addition, Fujian is a major destination for Taiwanese students and has the most diverse and multi-channel recruitment mechanisms for Taiwanese students among all provinces in mainland China. Fujian had opened 36 universities to recruit Taiwanese students, and full-time Taiwanese students in Fujian accounted for approximately one-sixth of the national total of Taiwanese students studying in mainland universities [7]. Therefore, given that Taiwanese students are dispersed across different provinces in mainland China, making it difficult to collect survey data broadly from multiple regions, this study selected Fujian as the research site.

Given the absence of a complete sampling frame for Taiwanese university students in Fujian Province, and considering that this population is difficult to access or even concealed, this study adopted a non-probability recruitment strategy combining convenience sampling and snowball sampling [46,47]. Participants were invited if they met the following three inclusion criteria: (1) they were aged 18 or above; (2) they identified as Taiwanese; and (3) they were Taiwanese students studying in Fujian Province, so as to control for the potential influence of geographic and cultural variation on the research results.

Recruitment was initiated through the Fujian Taiwanese Association, which then distributed the survey to its members. In parallel, the researcher disseminated the survey link through existing personal networks. To broaden the reach beyond single-network clusters, the survey link was also shared on three social media platforms commonly used by the target group, namely WeChat, Rednote, and Threads. All eligible participants were encouraged to forward the survey link to other qualified peers. These methods helped enhance the heterogeneity and ecological validity of the dataset [48].

The recruitment period spanned from October 29, 2025, to November 25, 2025, resulting in a total of 476 responses. To ensure the appropriateness and reasonableness of questionnaire data collection in this study, three screening criteria were adopted based on prior literature [49,50]. The standard screening criteria for valid questionnaires in this study were as follows: (1) a response time shorter than 50% of the expected completion duration; (2) duplicate IP addresses identified and verified through the Wenjuanxing platform (https://www.wjx.cn/); or (3) reverse-scored items within the same dimension display response patterns that are diametrically opposed to those of positively scored items. After excluding 51 invalid responses, a final sample of 425 valid questionnaires was retained for data analysis.

The minimum sample size was determined using G*Power software [51]. Because the structural model included a maximum of three predictors, the effect size was set at a medium level (0.15), with the statistical power at 0.80 and an alpha level of 0.05. The calculation indicated that the study required at least 77 respondents. Therefore, the final sample of 425 Taiwanese university students in Fujian was considered adequate for data analysis.

### 3.3. Common method bias

To rigorously address the potential common method bias (CMB) arising from the use of a self-report questionnaire, in addition to the procedural remedies of respondent anonymity, item randomization, and reverse coding, the full collinearity test was conducted. Following the recommendations of Kock [52–54], this study implemented the random dependent variable approach in SmartPLS to perform the full collinearity test. This procedure enables the detection of both vertical collinearity and lateral collinearity, thereby providing a more rigorous assessment of potential common method bias. The results showed that all variance inflation factor (VIF) values ranged from 1.153 to 2.815, which are below the recommended threshold of 3.3 [52], indicating that the results are unlikely to be contaminated by common method bias.

### 3.4. Sample profile

Table 1 presents the sample profile based on all valid responses. Female students accounted for the majority of the sample (57.4%). In terms of age, most Taiwanese university students were 26 years or younger (89.6%). With respect to educational level, the vast majority were undergraduates or below (80.7%). Regarding hometown, more than half of the participants were from the northern region of Taiwan (54.4%). As for the length of residence in mainland China, the majority reported living there for 10 years or more (59.1%).

**Table 1:** Respondents’ demographic profiles. (N = 425).

Category		Frequencies	Percentage
Age	18-22 years	310	72.9
	23-26 years	71	16.7
	27-35 years	41	9.6
	≥ 36 years	3	0.7
Gender	Male	181	42.6
	Female	244	57.4
Education Level	Diploma	80	18.8
	Bachelor	263	61.9
	Master	63	14.8
	Ph.D.	19	4.5
Hometown	Northern Region	231	54.4
	Central Region	96	22.6
	Southern Region	85	20
	Eastern Region	8	1.9
	Outlying Islands	4	0.9
	Other	1	0.2
Length of Residence	≥ 10 years	251	59.1
	< 10 years	174	40.9
Total		425	100

### 4. Results and findings

The current study employed partial least squares structural equation modeling (PLS-SEM) using SmartPLS 4 (v4.1.1.2) to estimate the proposed model. SEM is a commonly used variance-based approach and is particularly suitable for empirical research in the fields of management and the social sciences. The preference for PLS-SEM over covariance-based SEM (CB-SEM) is justified by specific methodological considerations. Although the model is grounded in the CTI, the specific interrelationships among PEIG, PRIG, and PCIG, along with their associations with psychological adaptation, require further clarification within the current research context, thereby prioritizing theory extension over pure theory testing. Furthermore, PLS-SEM is highly effective in addressing potential multicollinearity issues arising from conceptually similar predictors (e.g., PEIG, PRIG, and PCIG), thereby ensuring the robustness of the estimates [55,56]. Given that the primary objective is to maximize the in-sample explained variance (R²) of the endogenous constructs and considering PLS-SEM’s robustness as a non-parametric method, it serves as the appropriate statistical approach for this research [57].

### 4.1. Evaluating the measurement model

The measurement model was evaluated in two steps. First, reliability and convergent validity were established. Because all constructs in this study were represented by reflective indicators, and following Hair, all retained items showed factor loadings above the 0.60 threshold. All constructs demonstrated strong reliability, with Cronbach’s alpha and composite reliability (CR) values exceeding the 0.70 benchmark. Convergent validity was also confirmed, as all Average Variance Extracted (AVE) values exceeded the recommended 0.50 level. Second, discriminant validity was assessed using three approaches: the Heterotrait–Monotrait ratio (HTMT), the Fornell–Larcker criterion, and cross-loadings. Given that HTMT is more sensitive than traditional methods in detecting insufficient discriminant validity, this study treated it as the primary criterion. According to Henseler and Ringle, acceptable HTMT values should be below 0.90, while the more conservative threshold of 0.85 has also been widely applied. This study adopted the stricter threshold of 0.85 [58]. In addition, Fornell and Larcker [59] argued that discriminant validity is established when the square root of each construct’s AVE is greater than its correlations with other constructs. Furthermore, according to the recommendations of Chin [60] and Gefen and Straub [61], an indicator’s outer loading on its associated construct should be at least 0.10 higher than any of its cross-loadings on other constructs.

The final measurement model results are presented in Table 2. PRIG4 and PRIG6 were deleted because their factor loadings were below 0.60, and their removal could considerably enhance composite reliability or AVE [62]. Subsequent assessment of discriminant validity showed that discriminant validity between PCIG and PRIG was not supported under the conservative HTMT threshold of 0.85 (HTMT_PCIG–PRIG = 0.881), and the Fornell–Larcker criterion also indicated overlap (√AVE_PCIG = 0.786 < |r_PCIG,PRIG| = 0.809). Following the recommendation of Henseler et al. [63], this study retained the problematic constructs and proceeded to item-level diagnosis and refinement. Examination of the cross-loadings between PCIG and PRIG (see Table 3) showed that PCIG1 (loading on PCIG = 0.833; cross-loading on PRIG = 0.818; Δ = 0.015) and PCIG2 (loading on PCIG = 0.849; cross-loading on PRIG = 0.771; Δ = 0.078) showed insufficient separation from PRIG. At the same time, these items also showed conceptual overlap with PRIG. Therefore, reducing heterotrait–heteromethod correlations was the appropriate purification step for the current measurement model. After deleting PCIG1 and PCIG2, as shown in Table 4, all constructs met these criteria, thus establishing discriminant validity.

**Table 2:** Measurement model.

Construct	Items		Loadings	Cα	CR(c)	AVE
Personal-Enacted Identity Gap	PEIG1	0.821		0.890	0.917	0.653
	PEIG2	0.861				
	PEIG3	0.688				
	PEIG4	0.652				
	PEIG5	0.889				
	PEIG6	0.901				
Personal-Relational Identity Gap	PRIG1	0.857		0.921	0.940	0.759
	PRIG2	0.881				
	PRIG3	0.879				
	PRIG5	0.864				
	PRIG7	0.875				
Personal-Communal Identity Gap	PCIG3	0.756		0.847	0.892	0.623
	PCIG4	0.797				
	PCIG5	0.702				
	PCIG6	0.844				
	PCIG7	0.838				
Psychological Adaptation	PA1	0.650		0.887	0.909	0.559
	PA2	0.825				
	PA3	0.771				
	PA4	0.642				
	PA5	0.796				
	PA6	0.658				
	PA7	0.819				
	PA8	0.790				

Note: PRIG4 and PRIG6 were deleted (factor loading <0.6); PCIG1 and PCIG2 were deleted (cross loading <0.1); CR: composite reliability; AVE: average variance extracted.

**Table 3:** Cross loadings analysis.

	PA	PCIG	PEIG	PRIG
PA1	0.653	−0.614	−0.524	−0.502
PA2	0.825	−0.592	−0.539	−0.443
PA3	0.770	−0.446	−0.426	−0.331
PA4	0.639	−0.387	−0.358	−0.294
PA5	0.795	−0.522	−0.408	−0.401
PA6	0.656	−0.376	−0.307	−0.297
PA7	0.818	−0.527	−0.450	−0.388
PA8	0.793	−0.671	−0.566	−0.542
PCIG1	−0.521	0.833	0.663	0.818
PCIG2	−0.525	0.849	0.614	0.771
PCIG3	−0.608	0.684	0.499	0.471
PCIG4	−0.545	0.790	0.526	0.605
PCIG5	−0.602	0.621	0.526	0.409
PCIG6	−0.596	0.824	0.547	0.596
PCIG7	−0.550	0.866	0.592	0.709
PEIG1	−0.476	0.564	0.824	0.619
PEIG2	−0.524	0.644	0.864	0.641
PEIG3	−0.507	0.467	0.683	0.392
PEIG4	−0.475	0.470	0.645	0.392
PEIG5	−0.496	0.662	0.891	0.700
PEIG6	−0.531	0.669	0.903	0.723
PRIG1	−0.440	0.667	0.620	0.857
PRIG2	−0.452	0.698	0.613	0.882
PRIG3	−0.511	0.719	0.645	0.879
PRIG5	−0.475	0.702	0.634	0.864
PRIG7	−0.526	0.734	0.673	0.875

**Table 4:** Heterotrait-Monotrait analysis and Fornell & Larcker analysis.

Heterotrait-Monotrait criterion analysis					Fornell & Larcker criterion analysis			
	PA	PCIG	PEIG	PRIG	PA	PCIG	PEIG	PRIG
PA					0.748			
PCIG	0.827				−0.732	0.789		
PEIG	0.684	0.788			−0.617	0.683	0.808	
PRIG	0.591	0.801	0.793		−0.552	0.713	0.731	0.871

### 4.2. Evaluating the structural model

First, multicollinearity was assessed using the Variance Inflation Factor (VIF). According to Hair et al. [55], VIF values for the predictor constructs should be below 5 and preferably below 3. As shown in Table 5, all indicators had VIF values lower than 3, indicating that collinearity did not pose a threat to the analysis. Table 6 presents the baseline model path coefficients. Bootstrapping with 5,000 bias-corrected resamples was performed to assess the significance of the path coefficients and indirect effects [64]. Furthermore, the R² values were evaluated to determine the in-sample predictive power of the structural model [62].

**Table 5:** Multi-collinearity statistics.

	PA	PCIG	PEIG	PRIG
PA				
PCIG	2.296			
PEIG	2.425	2.146		1.000
PRIG	2.627	2.146		

**Table 6:** Results of hypothesis testing.

Hypothesis	Relationship	Path coefficient（β）	T statistics	P-values	Results
H1a	PEIG - > PA	−0.246	4.462	0.000	Supported
H1b	PRIG - > PA	0.061	1.007	0.314	Not Supported
H1c	PCIG - > PA	−0.608	11.850	0.000	Supported
H2a	PEIG - > PRIG	0.731	27.556	0.000	Supported
H2b	PEIG - > PCIG	0.349	7.142	0.000	Supported
H2c	PRIG - > PCIG	0.458	9.477	0.000	Supported
H3a	PEIG - > PRIG - > PA	0.044	1.006	0.314	Not Supported
H3b	PEIG - > PCIG - > PA	−0.212	6.506	0.000	Supported
H3c	PRIG - > PCIG - > PA	−0.278	6.830	0.000	Supported

**Fig 1:**
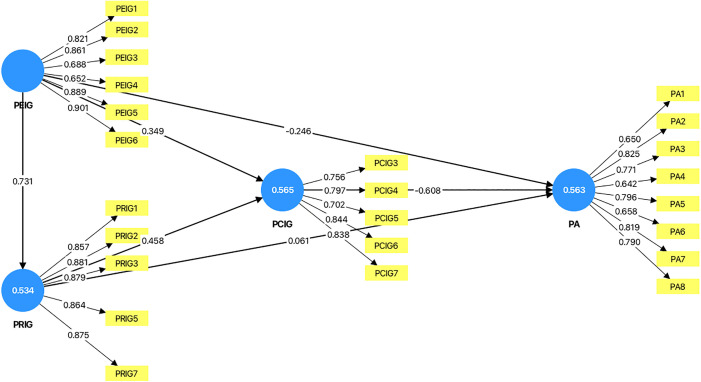
Baseline Model‌‌. Note: The values inside the circles represent the R² values.

As shown in Table 6 and Fig 1, the PEIG had a significant negative association with psychological adaptation (H1a: path coefficient = –0.246, t = 4.462, p < 0.001). In contrast, the association with the PRIG was not significant (H1b: path coefficient = 0.061, t = 1.007, p = 0.314). Consistent with expectations, the PCIG exerted a strong negative association with PA (H1c: path coefficient = –0.608, t = 11.850, p < 0.001). Regarding the interrelationships among the identity gaps, PEIG significantly associated with both PRIG (H2a: path coefficient = 0.731, t = 27.556, p < 0.001) and PCIG (H2b: path coefficient = 0.349, t = 7.142, p < 0.001). Similarly, PRIG was positively associated with PCIG (H2c: path coefficient = 0.458, t = 9.477, p < 0.001). For the mediation hypotheses, the indirect association between PEIG and psychological adaptation via PRIG was not significant (H3a: path coefficient = 0.044, t = 1.006, p = 0.314). However, PCIG significantly mediated the association of PEIG (H3b: path coefficient = –0.212, t = 6.506, p < 0.001) and PRIG (H3c: path coefficient = –0.278, t = 6.830, p < 0.001) with psychological adaptation.

Next, the explanatory power of the model was assessed. The R² value ranges from 0 to 1, with higher values indicating greater explanatory power. An R² above 0.100 is generally considered to reflect acceptable predictive power [65]. In the baseline model, the results revealed R² values of 0.534 for PRIG, 0.565 for PCIG, and 0.563 for PA, all of which were substantially higher than the 0.100 threshold (see Fig 1), thereby demonstrating the effective explanatory power capability of the model.

We also controlled for several confounding factors that might confound the variation in the main construct so as to avoid erroneous judgments. Based on a review of the literature on international students, we found that demographic variables such as age, gender, educational level, and length of residence have repeatedly appeared in studies of international students and may influence psychological adaptation [1,66]. Therefore, these control variables were incorporated into the baseline model to test the robustness of the model. Overall, the findings suggest that demographic variables play a limited role in explaining psychological adaptation, and the inclusion of these control variables does not materially alter the structural relationships, indicating strong model robustness. These analyses are described in detail in the S2 Appendix file.

**Fig 2:**
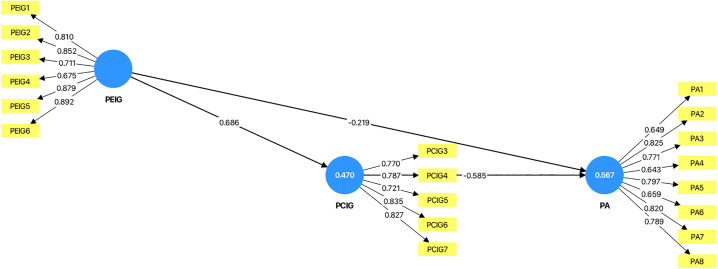
Alternative Model 1. (PRIG removed from the baseline model). Note: The values inside the circles represent the R² values.

The researchers further adjusted the model and tested two alternative models to examine the explanatory contribution of PRIG to psychological adaptation. In Alternative Model 1 (see Fig 2), when PRIG was removed from the baseline model, the R² value of PCIG decreased to 0.470, which represents only a minor reduction compared with the baseline model (0.565–0.470 = 0.095). The R² value of psychological adaptation increased slightly to 0.567, indicating no substantial change relative to the baseline model. After PRIG was removed, the results (see Table 7 and Fig 2) showed that both PEIG (path coefficient = –0.219, p < 0.001) and PCIG (path coefficient = –0.585, p < 0.001) remained significantly negatively associated with psychological adaptation. In addition, PEIG was positively associated with PCIG (path coefficient = 0.686, p < 0.001), and the indirect association between PEIG and psychological adaptation through PCIG was also significant (path coefficient = –0.401, p < 0.001).

**Table 7:** Path analysis results after removing PRIG from the model.

Relationship	Path coefficient（β）	T statistics	P values
PCIG - > PA	−0.585	13.081	0.000
PEIG - > PA	−0.219	4.450	0.000
PEIG - > PCIG	0.686	25.469	0.000
PEIG - > PCIG - > PA	−0.401	12.081	0.000

**Fig 3:**
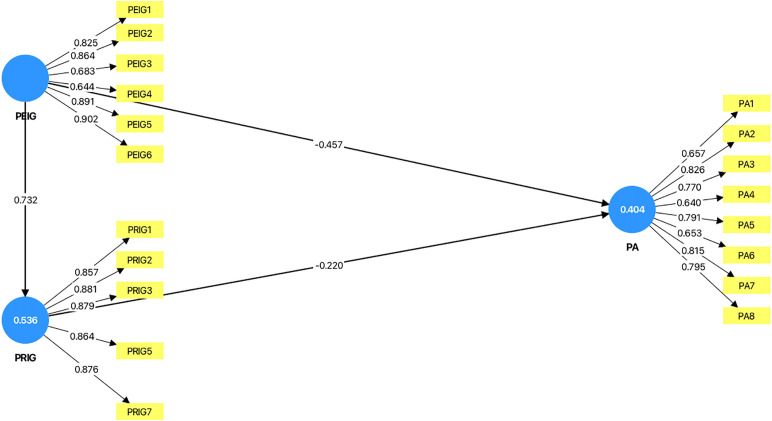
Alternative Model 2 (PCIG removed from the baseline model). Note: The values inside the circles represent the R² values.

In Alternative Model 2 (see Fig 3), when PCIG was removed from the baseline model, the R² value of psychological adaptation decreased to 0.404, representing a substantial reduction compared with the baseline model (0.565–0.404 = 0.161). After excluding PCIG, the results (see Table 8) showed that the direct association between PEIG and psychological adaptation remained significant. Notably, relative to the baseline model, PRIG was directly and negatively associated with psychological adaptation (path coefficient = –0.220, p < 0.001). In addition, the indirect association between PEIG and psychological adaptation through PRIG was also significant (path coefficient = –0.161, p < 0.001). These findings suggest that, in the absence of PCIG, PRIG emerges as a significant mediator in the association between PEIG and psychological adaptation.

**Table 8:** Path analysis results after removing PCIG from the model.

Relationship	Path coefficient（β）	T statistics	P values
PEIG - > PA	−0.457	7.774	0.000
PEIG - > PRIG	0.732	27.713	0.000
PRIG - > PA	−0.220	3.611	0.000
PEIG - > PRIG - > PA	−0.161	3.533	0.000

### 4.3. Importance–performance map analysis

**Fig 4:**
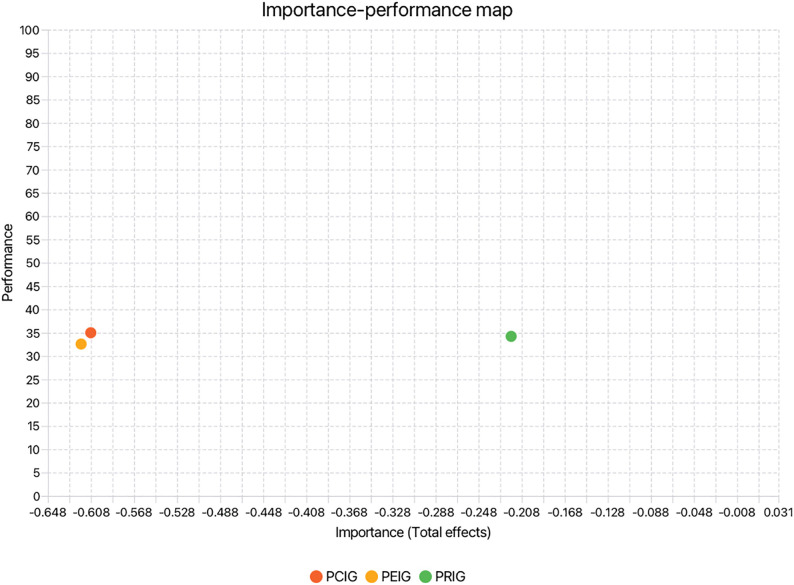
Importance-performance Map of the target psychological adaptation. (Baseline Model).

Finally, this study employed Importance–Performance Map Analysis (IPMA) (see Fig 4), which complements the PLS-SEM results by adding an additional analytical dimension that incorporates the average scores of the latent variables. This approach not only examines the total effects to determine the relative importance of each construct but also evaluates their average performance levels. The main objective of IPMA is to identify constructs that exert a strong overall influence on the outcome variable yet demonstrate relatively low performance, thereby indicating critical areas for potential improvement [67]. In this case (Fig 1 baseline model), the absolute importance values of PEIG and PCIG are high (PEIG = –0.617, PCIG = –0.608). This means that a one-unit increase in PEIG and PCIG would reduce psychological adaptation by up to 0.617 units and 0.608 units, respectively. The importance of PEIG and PCIG is substantially higher than that of PRIG, while the performance levels of the three constructs are relatively similar (PEIG = 32.577, PCIG = 35.004, PRIG = 34.223). Therefore, to enhance the performance of psychological adaptation, PRIG is the least critical construct and should be deprioritized.

## 5. Discussion

A stable identity is seen as a crucial factor for mobile populations during migration, with existing research linking identity gaps to adverse psychological outcomes. Nonetheless, despite the central role of identity, the associations between specific identity gaps and psychological adaptation remain insufficiently examined. Furthermore, there is still a lack of an integrated framework for conceptualizing the interrelationships among different identity gaps and identifying which of them are most closely associated with psychological adaptation, particularly in the context of intra-cultural migration within East Asia. To address these gaps, the present study draws on the CTI to empirically examine the associations between identity gaps and the psychological adaptation of Taiwanese university students in Fujian Province, China.

Regarding the direct associations, the hypotheses concerning the negative associations of PEIG, PRIG, and PCIG with psychological adaptation were only partially supported. A cultural perspective can further illuminate the significant negative association between PEIG and psychological adaptation. Suh [68] revisited the classical psychological proposition that identity consistency is a necessary condition for psychological well-being. Suh argued that, unlike North American societies that strongly emphasize consistency between internal consciousness and external behavior, East Asian cultures such as Korea place relatively less emphasis on behavioral consistency with internal thoughts. In such contexts, contradictions between identity and behavior may be perceived as reasonable or even necessary strategies for social interaction, where self-sacrificing compromises are viewed as important relational skills for preserving interpersonal harmony. This suggests that the association of PEIG with psychological adaptation in East Asian cultural settings may differ from that observed in Western contexts and that in some cases individuals may even actively tolerate or increase PEIG to achieve better well-being. However, Jung [35] found that among Korean immigrants in the United States, the personal–enacted identity gap directly and significantly predicted higher levels of depression, and this positive association was robust across different cross-group interactions. The present study is consistent with these findings, as PEIG was found to be significantly and negatively associated with the psychological adaptation of Taiwanese students in mainland China. One possible explanation is that, similar to Korean immigrants in the United States, Taiwanese youth, who have been shaped by Western cultural influences as well as democratic reforms and movements of individual emancipation [69], tend to reconstruct their personal identities with greater emphasis on expressing the authentic self. When this expression of authenticity is obstructed, their level of psychological adaptation is significantly diminished.

It is particularly noteworthy that the direct negative association of PEIG with psychological adaptation was substantially weaker than that of PCIG. This finding suggests that in East Asian societies that emphasize collectivism, the most intolerable inconsistency may be between personal identity and communal identity, and such a gap can severely hinder the psychological adaptation of Taiwanese students in mainland China. This result aligns with the cultural tradition in East Asia where individual values are largely dependent on collective evaluation. Several studies provide support for this view. Lin [70] pointed out that the psychological adaptation challenges faced by Taiwanese people in mainland China are largely due to their lower frequency of participation in communal activities with mainland friends, which reduces their sense of communal identification. Ju [71] found that participation in communal activities, particularly cultural and sports community events, can effectively enhance Taiwanese residents’ sense of happiness and integration. Xiao and Li [69] also argued that positive intergroup contact can foster greater cooperation among Taiwanese people in mainland China. Overall, the evidence suggests that communal identity may play a more prominent role than other identity layers in shaping the experiences of the Taiwanese population.

The hypotheses regarding the positive associations among PEIG, PRIG, and PCIG were all supported, which is consistent with prior research [15,41]. Particularly noteworthy is the finding that variance in PCIG was jointly explained by PEIG and PRIG. From a theoretical perspective, the presence of multiple antecedents of PCIG is understandable. Although the different layers are conceptually and diagnostically distinct [12,13,38], they are essentially interpenetrated and mutually influential [17,19]. For example, the communal identity layer is composed of multiple relational identities that share certain features, suggesting that changes in relational identity gaps may naturally extend to communal identity gaps. However, previous research has largely overlooked how identity gaps at smaller relational levels may be associated with the more macro-level PCIG. Scholars have also not fully acknowledged that the communal layer, which is formed through ascriptions at a broader societal level, may have stronger explanatory power for psychological outcomes such as adaptation and depression. Therefore, future research should pay greater attention to PCIG across different cultural contexts.

The hypotheses concerning the mediating roles of PRIG and PCIG in the negative association between PEIG and psychological adaptation were partially supported. This finding is consistent with several previous studies that reported non-significant results when PRIG was tested as an explanatory factor of negative psychological outcomes and behaviors [15,42,43,72]. Scholars have suggested two possible explanations for this pattern. First, the non-significant results of the two paths involving PRIG may be due to contradictions within the relational identity layer itself. Identity gaps do not only exist across different layers but may also occur within a single layer. For example, Kam and Hecht [72] argued that a young adult grandchild may identify as a “follower” when with the extended family (relational identity A) yet perceive themselves as a “leader” among peers (relational identity B). Such inconsistencies between different relational identities create within-frame identity gaps. The reason why PRIG is not negatively associated with psychological adaptation among Taiwanese students may lie in their possession of multiple, diverse, and sometimes contradictory relational identities. For instance, a required-course instructor may perceive a student as problematic, while a baseball coach may view them as the “best player” [73]. These diverse and potentially conflicting relational gaps may compete with each other, thereby reducing the explanatory power of PRIG for psychological adaptation. Future research should employ qualitative approaches, such as interviews, to further investigate the interplay within the relational identity layer and its connections with other layers. Second, the two alternative models in this study revealed an important finding: when both PRIG and PCIG are present, PCIG emerged as the central mediator and the more salient explanatory construct in relation to psychological adaptation. Comparisons of Alternative Model 1 and Alternative Model 2 with the baseline model in terms of regression coefficients and R² values show that, in models including PCIG as an explanatory construct, the explanatory capacity of PRIG may be subsumed by PCIG, and the inclusion or exclusion of PRIG does not materially alter the explained variance in psychological adaptation, which is consistent with the findings of our IPMA analysis. It is important to note that although the VIF, cross-loading, Fornell–Larcker, and HTMT criteria for each construct in this study met the required thresholds, two items from PRIG and two items from PCIG were deleted during testing because their factor loadings and cross-loadings did not meet the standards. This suggests that the measurement scales used in this study may still have limitations in terms of precision and discriminant validity when distinguishing between PRIG and PCIG. Future research could consider conceptualizing the two as a higher-order construct and further examining their relationships.

### 6. Conclusions and implications

The primary objective of this study is to investigate the influence of identity gaps on psychological adaptation in an Eastern cultural context. To achieve this goal, this study employed PLS-SEM, two alternative models, and PLS-IPMA for analysis. The results show that contractions in PEIG and PCIG are directly associated with improvements in psychological adaptation. The mediating effects of PRIG and PCIG were further scrutinized, with the findings identifying PCIG as the most consistently important explanatory construct.

### 6.1. Theoretical implications

Regarding its theoretical contributions, this study offers several important insights. Specifically, it expands the theoretical scope of CTI research. Previous studies have discovered that identity gaps can result in psychological outcomes, but they have yet to investigate the outcome variable of psychological adaptation. Therefore, by examining the mechanisms underlying the relationship between identity gaps and psychological adaptation among university students in mainland China and Taiwan, this study advances theory development.

Furthermore, previous research on non-local students has primarily focused on samples of international students in Western countries, especially the United States. This study is the first to apply identity gap theory to non-local student populations in East Asia, thereby revealing the cultural sensitivity of different types of identity gaps. The findings show that PEIG among Taiwanese university students in mainland China exhibits a negative association with outcome variables similar to that observed in Western cultural contexts. This result differs from the more general assumption that individuals in Eastern cultural settings may achieve better psychological outcomes through relatively implicit or restrained self-expression [68]. These findings suggest that, in addition to the inherent factors, such as the cultural backgrounds of Eastern and Western identities, the specific cultural context in which they were raised should also be taken into account when examining the effects of identity gaps.

Another important contribution lies in the emphasis on the role of PCIG in the process of psychological adaptation. Some scholars have questioned whether communal identity is necessary in studies of migrant populations because first-generation migrants may not yet have formed a stable communal identity framework [74,75]. In contrast, through alternative model comparisons and IPMA analysis, the present study demonstrates that PCIG is a key explanatory factor in understanding the psychological adaptation of Taiwanese students in mainland China.

In addition, this study clarifies the dynamic relationships among different identity gaps. Our findings suggest that the expansion of identity gaps at smaller relational levels may be associated with the widening of identity gaps at broader relational levels. By modeling PRIG and PCIG as mediators, we were able to capture how personal identity discrepancies originating at the level of PEIG, as the starting point of a discrete cycle of communicative misalignment, may be translated from an intrapersonal gap into more socially embedded interpersonal and communal misalignments. This finding lays an important foundation for future longitudinal research to clarify the developmental dynamics of identity gaps over time.

### 6.2. Practical implications

The findings of this research offer several practical implications for university administrators and policymakers seeking to enhance the psychological adaptation of Taiwanese students in mainland China.

A key implication of these findings is that university counseling centers should provide targeted psychological support to bridge identity gaps. Counselors should pay close attention to students’ identity negotiation dynamics. Specialized guidance is needed to help Taiwanese students develop a sense of psychological security, encouraging them to express their authentic selves in daily life rather than resorting to self-censorship. Creating a safe environment for self-expression is essential for reducing the intrapersonal tension.

Another important implication is that universities should foster nonpolitical intergroup contact on campus. To address the lower levels of community participation observed among Taiwanese students [8], administrators should design and encourage involvement in collective activities, such as cultural programs, sports events, and volunteer services. These interactions with mainland Chinese peers serve as a vital platform for reducing mutual misunderstandings and fostering a stronger sense of communal belonging [76,77].

At the broader policy level, policymakers should expand field research and study tour programs to dismantle deep-seated stereotypes. Research indicates that negative media narratives often foster pre-existing prejudices and a sense of alienation among Taiwanese students, while mainland residents may also hold inaccurate perceptions due to limited contact. Previous studies have shown that when Taiwanese students conduct field visits in mainland China, their satisfaction increases, social distance decreases, and even their national identification is significantly enhanced [78–80]. Therefore, government and relevant institutions should continue to strengthen policies that facilitate broad exposure to different regions of mainland China, specifically by organizing regular field visits and social investigations.

### 6.3. Limitations

This study has several limitations that point to directions for future research. First, due to time and budget constraints, the cross-sectional survey was conducted only in the Fujian Province of mainland China. The findings may not fully represent Taiwanese students across other provinces. However, collecting a large-scale sample from all 31 provincial-level administrative divisions is challenging because the number of Taiwanese students is relatively small in many provinces, and they are dispersed across different universities. Future research may attempt to collect data from a wider range of provinces, which would provide a more comprehensive picture of Taiwanese students’ adaptation in mainland China. Second, in terms of research methods, both qualitative and quantitative approaches are suitable for the study of identity gaps [40,81]. The present study relied solely on PLS-SEM for quantitative analysis, which may have limited the scope of understanding psychological adaptation among Taiwanese students in mainland China. Future work could employ mixed methods to capture a fuller picture. Third, most quantitative studies on international students’ adaptation have used cross-sectional designs, with only a few longitudinal investigations [82]. While existing research often suggests that identity gaps predict psychological outcomes, some studies have instead treated psychological factors as antecedent variables that influence the formation of identity gaps [11,13]. Future research should therefore prioritize longitudinal designs to clarify the directionality of these relationships.

Finally, this study considered identity gaps only as independent variables. However, identity gaps are themselves shaped by multiple antecedent factors [40]. Therefore, the present research may not fully reflect the complex challenges faced by Taiwanese students in mainland China. For example, a promising direction for future research is to examine how online communication influences the identity gaps of Taiwanese university students, who are digital natives, and subsequently affects their psychological adaptation.

## Supporting information

S1 Appendix Scale items. (DOCX)

S2 Appendix Supplementary analyses: Control variables. (DOCX)
