# Supplementary material for: Contracting the gap: The effects of identity gaps on the psychological adaptation of Taiwanese university students
Source: PLoS One. 2026 May 19;21(5):e0349289. doi: 10.1371/journal.pone.0349289 (PMC13186331; doi:10.1371/journal.pone.0349289)
Supplement: S1 Appendix — (DOCX) [file pone.0349289.s001.docx]

| **Construct** | **Code** | **Item** |
| --- | --- | --- |
| Personal-Enacted Identity Gap (PEIG) | PEIG1 | I usually communicate with my friends from Chinese mainland in the way that is consistent with who I really am. (R) |
|  | PEIG2 | I feel I can be myself when communicating with my friends from Chinese mainland. (R) |
|  | PEIG3 | I often hide some aspects of myself in communication with my friends from Chinese mainland. |
|  | PEIG4 | I feel there are differences between the “real me” and the impressions I give my friends from Chinese mainland. |
|  | PEIG5 | I speak truthfully to my Chinese mainland friends about myself. (R) |
|  | PEIG6 | I freely express the “real me” in communication with my friends from Chinese mainland. (R) |
| Personal-Relational Identity Gap (PRIG) | PRIG1 | I usually agree with what my Chinese mainland friends describe about me. (R) |
|  | PRIG2 | I think my Chinese mainland friends see me as how I see myself. (R) |
|  | PRIG3 | I feel my Chinese mainland friends have correct images of me. (R) |
|  | PRIG4 | I feel my Chinese mainland friends stereotype me. |
|  | PRIG5 | I think my Chinese mainland friends know what kind of person I am. (R) |
|  | PRIG6 | I feel there are differences between who I think I am and who my Chinese mainland friends think I am. |
|  | PRIG7 | I feel my Chinese mainland friends have correct information about me. (R) |
| Personal-Communal Identity Gap (PCIG) | PCIG1 | I feel that how the community of my Chinese mainland friends see me matches well with how I see myself. (R) |
|  | PCIG2 | What I expect from a community matches what is provided by the community of my Chinese mainland friends. (R) |
|  | PCIG3 | I feel like an outsider in the community that my Chinese mainland friends belong to. |
|  | PCIG4 | I feel proud to be a member of the community that my Chinese mainland friends belong to. (R) |
|  | PCIG5 | I feel embarrassed that I am part of the community that my Chinese mainland friends belong to. |
|  | PCIG6 | I feel like I belong in the community of my Chinese mainland friends. (R) |
|  | PCIG7 | The way I see the members in the community of mainland Chinese is the same as how I see myself. (R) |
| Psychological Adjustment (PA) | PA1 | I feel excited about being in Chinese mainland. |
|  | PA2 | I fell out of place when I don’t fit into the culture of Chinese mainland (R). |
|  | PA3 | I feel sad to be away from Taiwan (R). |
|  | PA4 | I feel nervous about how to behave in certain situations (R). |
|  | PA5 | I feel lonely without my Taiwan family and friends around me (R). |
|  | PA6 | I feel homesick when I think of Taiwan (R). |
|  | PA7 | I feel frustrated by the difficulties of adapting to the life on Chinese mainland (R). |
|  | PA8 | I feel happy with my day-to-day life on Chinese mainland. |

[**S1.**](https://journals.plos.org/plosone/article/file?type=supplementary&id=10.1371/journal.pone.0212615.s001)**Scale items.**

Note: The (R) in the table indicates reverse-coded items.
