# Supplementary material for: Contracting the gap: The effects of identity gaps on the psychological adaptation of Taiwanese university students
Source: PLoS One. 2026 May 19;21(5):e0349289. doi: 10.1371/journal.pone.0349289 (PMC13186331; doi:10.1371/journal.pone.0349289)
Supplement: S2 Appendix — (DOCX) [file pone.0349289.s002.docx]

**S2. Supplementary Analyses: Control variables**

In the questionnaire, respondents were asked to report their age, gender, and educational level (Diploma = 1, Bachelor = 2, Master = 3, Ph.D. = 4), and length of residence (≥ 10 years = 1, < 10 years = 2). To assess the effects of the control variables, a bootstrapping procedure with 5,000 resamples was conducted in SmartPLS 4.

The results (Table S1) indicate that age has a statistically significant negative effect on psychological adaptation (path coefficient = −0.110, t = 2.189, p = 0.029), suggesting that older individuals tend to report lower levels of psychological adaptation. In contrast, gender (path coefficient = −0.115, t = 1.803, p = 0.071), educational level (path coefficient = 0.075, t = 1.597, p = 0.110) and length of residence (path coefficient = 0.108, t = 1.660, p = 0.097) do not exhibit statistically significant effects at the 0.05 level. At the same time, the extended model (Fig S1) shows that the control variables added to the baseline model have only a very limited impact on the path coefficients and R² values of the baseline model. This further supports the proposition that, among Taiwanese university students studying in mainland China, identity gaps constitute a fundamental explanatory mechanism underlying psychological adaptation, independent of most demographic influences.

**Table S1. Control variables.**

| **Relationship** | **Path coefficient（β）** | **T statistics** | **P values** |
| --- | --- | --- | --- |
| education- > PA | 0.075 | 1.597 | 0.110 |
| gender-> PA | -0.115 | 1.803 | 0.071 |
| Length of Residence-> PA | 0.108 | 1.660 | 0.097 |
| age -> PA | -0.110 | 2.189 | 0.029 |





**Fig. S1 Extended Model.**

Note: The values inside the circles represent the R² values.
